# Experiences of Self‐Sampling and Future Screening Preferences in Non‐Attenders Who Returned an HPV Vaginal Self‐Sample in the YouScreen Study: Findings From a Cross‐Sectional Questionnaire

**DOI:** 10.1111/hex.14118

**Published:** 2024-07-02

**Authors:** Hannah Drysdale, Laura A. V. Marlow, Anita Lim, Jo Waller

**Affiliations:** ^1^ Cancer Prevention Group, School of Cancer and Pharmaceutical Sciences, King's College Faculty of Life Sciences and Medicine London UK; ^2^ Centre for Cancer Prevention, Screening and Early Diagnosis, Wolfson Institute of Population Health, Faculty of Medicine and Dentistry Queen Mary University of London London UK

**Keywords:** cervical screening, confidence, HPV vaginal self‐sampling, screening non‐attenders, screening preferences, trust

## Abstract

**Background:**

We assessed experiences of human papillomavirus (HPV) vaginal self‐sampling and future screening preferences in an ethnically and socio‐economically diverse group of women overdue for cervical screening.

**Setting and Participants:**

A postal questionnaire was embedded in the YouScreen self‐sampling trial in England: 32.5% (2712/8338) of kit completers returned the survey. Kit non‐completers were encouraged to return a questionnaire, but no responses were received. Participants were ethnically diverse (40.3% came from ethnic minority backgrounds), and 59.1% came from the two most deprived quintiles. Differences in confidence in kit completion, trust in the test results and intention to attend a follow‐up test if HPV‐positive were evaluated using Pearson's *χ* ^2^ analyses. Binary logistic regression models explored predictors of a future screening choice and preferences for urine versus vaginal self‐sampling.

**Results:**

Most kit‐completers reported high confidence in self‐sampling (82.6%) and high trust in the results (79.9%), but experiences varied by ethnicity and screening status. Most free‐text comments were positive but some reported difficulties using the device, pain or discomfort. Most women would opt for self‐sampling in the future (71.3% vs. 10.4% for a clinician‐taken test) and it was more often preferred by ethnic minority groups, overdue screeners and never attenders. Urine self‐tests were preferred to vaginal tests (41.9% vs. 15.4%), especially among women from Asian, Black or Other Ethnic backgrounds.

**Conclusions:**

Kit‐completers were confident, found the test easy to complete, and trusted the self‐sample results. However, experiences varied by ethnic group and some women highlighted difficulties with the kit. Most women would prefer self‐sampling in the future, but it was not a universal preference, so offering a choice will be important.

**Patient or Public Contribution:**

We did not have direct patient and public involvement and engagement (PPIE) in the questionnaire design. However, patients and public representatives did input into the design of the YouScreen trial and reviewed the wider study materials (e.g. participant information sheet).

**Trial Registration:**

This questionnaire study was embedded in the YouScreen trial. The protocol for the YouScreen trial is available at https://www.isrctn.com/ISRCTN12759467. The National Institute for Health Research 43 Clinical Research Network (NIHR CRN) Central Portfolio Management System (CPMS) ID is 4441934.

## 1 Introduction

In England, women aged 25−64 years are offered a cervical screening test every 3 or 5 years, depending on their age. Evidence shows that ~70% of cervical cancer deaths are prevented by screening [1], but uptake is at an all‐time low. Cervical screening coverage has declined over the last decade, and ~30% of women are now estimated to be overdue [2]. Screening non‐attenders are at greater risk of developing cervical cancer [1], but evidence shows that self‐sampling, which has been facilitated by the introduction of HPV primary screening, can help increase uptake in these women [3, 4, 5, 6].

HPV self‐sampling requires an individual to collect their own sample before returning it to a laboratory for testing. There are several self‐sampling approaches available, but most evidence to date focuses on vaginal self‐sampling using a long swab or soft brush. There is mounting evidence that HPV self‐sampling, with clinician testing for cytology triage in HPV‐positive women, is an acceptable and cost‐effective alternative to clinician‐taken screening and has a similar level of sensitivity to detect high‐grade disease [3, 7]. Self‐sampling also empowers women to take control of their screening experience and potentially addresses known barriers to a speculum examination including discomfort, embarrassment and inconvenient appointment times [8, 9, 10, 11]. Despite its appeal, some research suggests women have reservations about the test, notably, a lack of confidence in collecting the sample themselves and concerns over test accuracy [12, 13, 14, 15]. Clear step‐by‐step instructions and the opportunity to ask healthcare professionals questions, can help reassure women that they are collecting a sample correctly [13]. There is also emerging evidence that HPV urine self‐sampling could be an alternative future test, which may appeal to women who do not find vaginal collection acceptable. Existing research suggests that women have greater confidence in providing a urine sample and a larger proportion would prefer this type of self‐test (compared to a vaginal kit) in the future [16, 17].

The effectiveness of self‐sampling (both in terms of cost and lives saved) also depends on high adherence to follow‐up for a clinician‐taken test if women test positive for HPV [18]. This additional visit to a clinician is required so that a cervical sample can be collected for cytology triage. Adherence to follow‐up is estimated to be around 80%, but there is heterogeneity across trials (range: 41%−100%) and uptake varies between screening non‐attenders and regular attenders [3].

HPV vaginal self‐sampling has already been integrated into other national screening programmes. In some countries it has been used as strategy to target non‐attenders (e.g., Denmark and Finland) and in others it is part of a choice‐based offer (e.g., Australia, the Netherlands and Sweden) [19, 20, 21, 22]. Research studies suggest that self‐sampling can help to increase cervical screening uptake in non‐attenders, but it varies depending on the type of offer (5.2%−10.9% for opt‐in strategies vs. 6.4%−34.0% for mailed kits) [3]. Data that has been collected from countries that have already implemented self‐sampling paints a less encouraging picture, suggesting that it may not have had the anticipated impact on screening coverage [23]. Self‐sampling is yet to be rolled out in England, but the National Health Service (NHS) cervical screening programme has stated its intention to carry out a UK‐wide in‐service evaluation which will offer a choice of vaginal self‐collection or standard clinician sampling to all invitees [24]. This would assess the feasibility of offering a choice of self‐sampling within the UK screening pathway.

The YouScreen pragmatic implementation feasibility trial was the first English study to integrate HPV vaginal self‐sampling into the NHS cervical screening programme. The trial, which ran in 2021, invited over 27,000 women who were overdue for screening to complete a self‐sampling kit. Women aged between 25 and 64 years were recruited from 133 GP practices in London [25]. This acceptability substudy was nested within the trial. We aimed to answer the following questions: How much confidence and trust did women in YouScreen have in the self‐sampling test? How likely did they say they were to attend for a follow‐up clinician‐taken test if they were HPV positive? What future screening choices would these women make after completing a vaginal self‐sampling kit? And how did experiences and preferences vary by demographic groups? This was the first study in England to explore self‐sampling experiences and preferences following a real‐world offer and included detailed information on women's screening history, screening behaviours, ethnicity and level of deprivation. The results provide evidence for the future planned evaluation of self‐sampling in England and contribute to worldwide evidence on the acceptability of HPV self‐sampling.

## 2 Methods

### 2.1 The YouScreen Trial

This questionnaire study was embedded in the YouScreen trial (see [25] for full methodological details). In brief, the trial recruited GP practices across five urban London boroughs (Camden, Islington, Tower Hamlets, Newham and Barnet), which had consistently low cervical screening coverage. Women were offered a self‐sampling kit opportunistically when they presented in primary care if they were ≥ 6 months overdue (‘opportunistic’ offer) or were sent a kit in the post if they reached 15 months overdue since the invitation (‘mailed’ offer). GPs, nurses and healthcare assistants received training on the opportunistic offer as part of site initiation visits. As part of this training, they were provided with key messages to communicate (e.g., ‘the practice is offering self‐sampling kits as part of a research study that aims to make screening easier for women’). The training video, which includes suggested scripts for the consultation, can be accessed on YouTube (https://www.youtube.com/watch?v=Yd-G6KcBEmw). Healthcare professionals were also signposted to online resources, such as FAQs, which discussed how self‐sampling should be brought up in consultations and whether kits could be offered via virtual or telephone appointments (for further information, please visit: https://www.nclcanceralliance.nhs.uk/our-work/primary-care-2/gp-info-youscreen). Women received their screening results by letter, in line with the existing cervical screening pathway. Those who tested positive for HPV were advised in their results letter to attend their GP surgery for a follow‐up clinician‐taken test.

The self‐sampling kit included a flocked swab (FLOQswab™, 552c.80 Copan Italia), completion instructions (written and pictorial), a participant information booklet, a laboratory request consent form, a pre‐paid envelope to return their sample and the substudy questionnaire. Participant‐facing materials were designed to help bolster women's confidence in completing the kit, and women were able to access additional resources for support (e.g., an instructional video hosted on the study's microsite [www.smallc.org.uk/get-involved/get-involved-youscreen/], a dedicated helpline and the information booklet was translated into five different languages).

### 2.2 Design

The present study was a cross‐sectional paper‐based survey that was included in the self‐sampling kit (described above). The questionnaire was printed on a single page of A4 paper, folded and self‐sealed with pre‐paid postage to King's College London. Trial participants were asked to complete and post the questionnaire separately to their self‐sample. The questionnaire and pre‐registered statistical analysis plan are available on Open Science Framework (https://osf.io/b3m6h/).

### 2.3 Participants

Inclusion criteria were established as part of the broader trial. Women aged ≥ 25.5 and ≤ 64 years, eligible for cervical screening, at least 6 months overdue for their test, and registered at a participating GP practice were included. Women aged 65 years were not considered to be a violation of the protocol and were included in analyses. These women had either not met the threshold to be ceased from the programme or were being offered a test because they had not attended since the age of 50.

The sample size was dictated by the main trial, but our a priori expectations were that 31,000 kits, including questionnaires, would be issued. We anticipated a 90% response rate for kit completers and 10% for kit non‐completers based on a previous self‐sampling study in Westminster, London [26]. This was expected to give us a sample of 3891 responses based on a 20% kit completion rate for women who were mailed a kit and 30% for the opportunistic offer.

Ethical approval for the trial (National Institute for Health Research [NIHR] ID: 41934) was granted by the South Birmingham Research Ethics Committee (20/WM/0120), IRAS (ID 264776), the Confidentiality Advisory Group (20/CAG/0086) and the CSP Research Advisory Committee (CSP‐RAC‐032).

### 2.4 Measures

#### 2.4.1 Confidence and Trust

Participants were asked how confident they were that they had completed the kit correctly and how much they trusted the results of the test. These were single items with 5‐point response scales, anchored at each end (e.g., 1 [not at all confident] to 5 [very confident]). For analyses, we recoded confidence and trust into binary variables, following a visual inspection of the distribution of responses (‘high’ confidence or trust [4 or 5 rating] and ‘low’ confidence or trust [1, 2 or 3 rating]). Women were also asked if they had any comments or concerns about completing the kit or results (a free‐text response question).

#### 2.4.2 Intention to Attend Follow‐Up

Follow‐up intentions were assessed by asking how likely participants were to go for follow‐up cervical screening if their result was HPV positive. Responses were recoded into three groups because they showed a bimodal distribution: ‘high likelihood’ (1 rating), ‘mid‐likelihood’ (2, 3 or 4 rating) and ‘low likelihood’ (5 rating).

#### 2.4.3 Future Preferences for Screening

##### 2.4.3.1 Preferred Screening Method

Having completed a vaginal self‐sampling kit, women were asked for their preferred method of future screening (*‘In the future, would you prefer to do a self‐sample or have a health professional do the test?*’). Response options were ‘prefer self‐sampling’, ‘prefer a health professional to do the test’ (clinician screening) or ‘no preference’. At this point in the survey, the concept of urine sampling had not been mentioned, so we interpret the selection of ‘self‐sampling’ to refer to vaginal self‐sampling as used in YouScreen.

##### 2.4.3.2 Preferences for Urine or Vaginal Self‐Sampling

Women were invited to select their preferred type of self‐sampling test if a urine test were available. Response options were ‘a urine self‐sample’, ‘a vaginal self‐sample like YouScreen’ or ‘no preference’.

##### 2.4.3.3 Preference for Receiving a Self‐Sampling Kit

Women were asked how they would prefer to receive a self‐sampling kit in the future. Response options were ‘in the post’, ‘in person at your GP surgery’ or ‘no preference’.

##### 2.4.3.4 Preference for Receiving Results

Women indicated whether they would be willing to receive their screening results by ‘text message’, ‘email’ and ‘letter’ (they could tick multiple options).

#### 2.4.4 Sociodemographics

##### 2.4.4.1 Age and Ethnicity

In the questionnaire, women were asked to self‐report their age and ethnicity. We also imported age and ethnicity information from GP electronic record data that had been extracted for the main trial to help with missing data. Self‐reported information took precedence if there were discrepancies. Age was collapsed into 10‐year age bands and ethnicity was collapsed into smaller groups for analysis (see Table 1).

##### 2.4.4.2 Socioeconomic Status

The index of multiple deprivation 2019 (IMD), a national measure of relative deprivation, was used as an area‐level proxy for socioeconomic status. Women's postcodes in NHAIS (National Health Application and Infrastructure Services—the national screening database) were converted to IMD deciles using Lower Layer Super Output Areas from the 2019 English indices of deprivation report. We recorded IMD deciles into quintiles for analysis.

##### 2.4.4.3 Screening Status

This variable was created using imported line‐level data from NHAIS screening records, which gave the date of each woman's last adequate NHS screening test (before the study start date). ‘Overdue’ screeners were defined as women who were up to 24 months overdue for their screening test based on their last due date and ‘very overdue’ screeners were defined as those who were over 24 months overdue. Women defined as never attenders had on no occasion attended a cervical screening test. Women that were aged 25−27 years with no recorded screening tests were categorised as ‘overdue’.

##### 2.4.4.4 Mode of Distribution

This variable was based on the first type of kit that was received at the laboratory (the self‐sampling kit numbers indicated the mode of kit distribution). Women were categorised as ‘opportunistic’ or ‘mailed’ depending on how they had received their self‐sampling kit.

### 2.5 Analysis

All analyses were conducted using IBM SPSS Statistics Version 29.0. A conservative significance level of *p* < 0.01 was applied to account for multiple comparisons.

The proportion of participants who selected each response option for confidence, trust and intention to follow‐up and the proportion who selected each preferred future screening method (self‐sampling vs. clinician‐screening and a urine test vs. a vaginal test) was reported overall and by the sociodemographic group. Unadjusted logistic regression models were used to identify significant differences in experiences of self‐sampling between demographic groups. We compared those who indicated a high versus low level of confidence/trust and the proportion who intended to attend a follow‐up test (high intention vs. mid and low intentions).

Two binary multivariable logistic regressions explored the associations between demographic predictors and future preferences for self‐sampling. The first model compared a self‐sampling choice to a clinician screening choice or no preference. The second model compared preferences for urine self‐sampling to vaginal collection or no preference. An a priori decision was taken to include all predictor variables (age, ethnicity, socioeconomic status, screening status and mode of distribution) in the model.

Content analysis was used to code women's responses to the free‐text question into themes. A coding framework was inductively developed before two authors (H. D. and L.M.) independently coded the data. Multiple codes could be allocated to a single comment. Any discrepancies were resolved through discussion. Themes were reported descriptively (the percentage of women endorsing the theme as a total of all women that provided a comment).

## 3 Results

### 3.1 Participant Characteristics

Out of a total of 8338 women who completed a self‐sampling kit in the YouScreen trial, 2712 returned a questionnaire (32.5% response rate). Fifty‐eight responses were excluded (*n* = 6 duplicate questionnaires [indicating responses from an opportunistic and a mailed kit], *n* = 4 were ineligible and *n* = 48 had not answered any of the 22 survey questions—excluding age, ethnicity and free‐text questions). This left 2654 responses for analysis. Although we encouraged those not completing a kit to fill in the questionnaire, we did not receive any responses from this group.

Table 1 shows the demographic characteristics of questionnaire responders. The mean age was 40.8 years (range of 2565 years), 74.3% were eligible for 3‐yearly screening intervals (25−49 years) and 25.7% were eligible for 5‐yearly intervals (50−65 years). Responders were ethnically diverse (40.3% were from ethnic minority backgrounds) and 59.1% lived in the two most deprived quintiles. A similar proportion of women had received their self‐sampling kit opportunistically (50.8%) or via the post (49.2%). Half of kit‐completers were identified as overdue screeners (up to 24 months late for their test) (49.5%) and around a quarter were either very overdue (over 24 months late for their test) or had never previously attended screening (23.2% and 27.0%, respectively).

**Table 1:** Demographic characteristics of questionnaire responders and YouScreen kit completers.

Type of responder	Questionnaire responders (*n* = 2654)		All YouScreen kit completers (*n* = 8338)	
	*n*	%	*n*	%
*Age*				
25−34 years	1005	37.9	3198	38.3
35−44 years	691	26.0	2257	27.1
45−54 years	536	20.2	1552	18.6
55−65 years	422	15.9	1331	16.0
*Ethnicity*				
White (English, Welsh, Scottish, N. Irish, British)	675	25.5	1531	18.3
White ‐ Other (Irish and Other)	802	30.2	807	9.7
Mixed/multiple ethnic groups	112	4.2	1176	14.1
Asian or Asian British	614	23.1	1905	22.9
Black, Black British, Caribbean or African	182	6.9	668	8.0
Other ethnic group	162	6.1	339	4.1
Prefer not to say or missing	107	4.0	1912	22.9
*Socioeconomic status (IMD)*				
Q1 (most deprived)	463	17.5	1598	19.2
Q2	1105	41.6	3383	40.6
Q3	564	21.3	1640	19.7
Q4	319	12.0	1117	13.4
Q5 (least deprived)	195	7.3	548	6.5
Missing	8	0.3	52	0.6
*Mode of distribution*				
Mailed	1307	49.2	2277	27.3
Opportunistic	1347	50.8	6061	72.7
*Screening status*				
Overdue (up to 24 months since last due date)	1313	49.5	3973	47.7
Very overdue (> 24 months since last due date)	617	23.2	2020	24.2
Never attended	716	27.0	2296	27.5
Missing	8	0.3	49	0.6

Questionnaire responders were similar to YouScreen kit completers in respect of age, socioeconomic status and screening status. However, a smaller proportion of questionnaire responders came from Mixed or Other Ethnic backgrounds (10.3% vs. 18.2% for YouScreen). In contrast, a larger proportion identified as White (55.7% vs. 28.0% for YouScreen) and came from the mailed arm (49.2% vs. 27.3% for YouScreen).

### 3.2 Study Outcomes ‐ Experiences of Self‐Sampling

**Figure 1:**
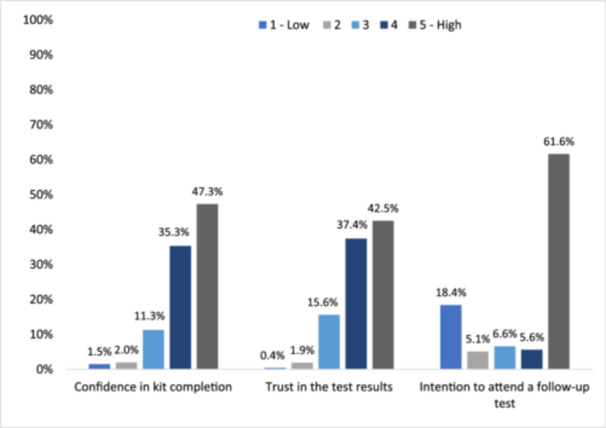
Experiences of self‐sampling (*n* = 2654) (%).

Most women reported high confidence in kit completion (82.6%) and high trust in the results (79.9%). A considerable proportion had high intentions of going for a follow‐up cervical screening test if HPV was found (61.6%), 17.3% reported a medium likelihood, and 18.4% self‐reported low intentions (Figure 1).

Although confidence and trust were high across demographic subgroups, there were some significant differences. Asian women were less likely to report high confidence compared to women from a White British background (unadjusted odds ratio [OR] = 0.5, 95% CI: 0.4−0.7) and were less likely to trust the results of the test (OR = 0.7, 95% CI: 0.5−0.9). In contrast, those who had never been screened were more likely to report high trust in the results compared to overdue screeners (OR = 1.6, 95% CI: 1.3−2.1). There were also differences in intention to attend a follow‐up. Asian women (OR = 0.7, 95% CI: 0.6−0.9) and those from Other Ethnic groups (OR = 0.6, 95% CI: 0.4−0.9) reported lower intentions than White British women and very overdue screeners were less inclined to attend a clinician‐taken test compared to overdue screeners (OR = 0.7, 95% CI: 0.6−0.9) (Table 2). There were no significant differences by age, socioeconomic status or mode of distribution.

**Table 2:** Associations between sociodemographic characteristics and experiences of self‐sampling.

	High confidence in completing the kit (rating of 4/5 on the scale)			High trust in the test results (rating of 4/5 on the scale)			High intentions to attend a follow‐up test (rating of 1 on the scale)		
Experiences of self‐sampling	%	*n*	OR	%	*N*	OR	%	*n*	OR
*Age*									
25−34 years	85.1	987	Ref	82.3	992	Ref	63.1	987	Ref
35−44 years	85.6	674	1.04 (0.79−1.37)	81.2	677	0.93 (0.73−1.20)	64.0	670	1.04 (0.85−1.28)
45−54 years	84.4	520	0.95 (0.71−1.27)	82.6	522	1.02 (0.77−1.35)	65.1	521	1.09 (0.87−1.36)
55−65 years	83.0	406	0.86 (0.63−1.17)	79.8	406	0.85 (0.64−1.14)	60.3	406	0.89 (0.70−1.13)
*Ethnicity*									
White	88.3	659	Ref	83.9	665	Ref	68.2	666	Ref
White ‐ Other	86.1	787	0.82 (0.60−1.12)	85.0	788	1.09 (0.82−1.45)	64.6	793	0.85 (0.68−1.06)
Mixed/multiple ethnic groups	87.5	112	0.93 (0.50−1.70)	84.5	110	1.05 (0.60−1.83)	62.7	110	0.79 (0.52−1.20)
Asian	80.1	594	0.53 (0.39−0.73)*	77.9	598	0.68 (0.51−0.90)*	59.7	591	0.69 (0.55−0.87)*
Black	83.5	176	0.67 (0.42−1.07)	79.5	176	0.75 (0.49−1.14)	60.7	173	0.72 (0.51−1.02)
Other ethnic group	84.2	158	0.70 (0.43−1.15)	79.9	159	0.76 (0.49−1.18)	56.7	157	0.61 (0.43−0.87)*
*Socioeconomic status (IMD)*									
Quintile 1 (most deprived)	82.4	449	1.02 (0.66−1.59)	80.0	450	0.71 (0.45−1.13)	61.1	447	0.72 (0.50−1.03)
Quintile 2	84.5	1080	1.19 (0.79−1.79)	82.0	1080	0.81 (0.53−1.24)	61.3	1066	0.73 (0.52−1.01)
Quintile 3	85.9	548	1.33 (0.86−2.08)	81.3	552	0.78 (0.50−1.22)	67.0	551	0.93 (0.65−1.32)
Quintile 4	88.8	313	1.73 (1.04−2.89)	81.9	315	0.81 (0.49−1.31)	63.8	318	0.81 (0.55−1.18)
Quintile 5 (least deprived)	82.1	190	Ref	84.9	192	Ref	68.6	194	Ref
*Mode of distribution*									
Mailed	84.8	1279	Ref	80.3	1277	Ref	61.3	1275	Ref
Opportunistic	84.8	1308	1.00 (0.81−1.24)	83.0	1320	1.20 (0.99−1.47)	65.3	1309	1.19 (1.02−1.40)
*Screening status*									
Overdue (up to 24 months since due date)	84.1	1284	Ref	79.0	1286	Ref	65.6	1289	Ref
Very overdue (> 24 months since due date)	86.0	599	1.16 (0.88−1.53)	82.9	601	1.29 (1.00−1.65)	58.7	595	0.74 (0.61−0.91)*
Never attended	85.1	697	1.08 (0.83−1.39)	85.8	702	1.60 (1.25−2.06)*	63.0	692	0.89 (0.74−1.08)

*Note:* Denominators in the table vary between items due to missing questionnaire data.

Abbreviationsc: OR, unadjusted odds ratio; Ref, reference group.

* *p *< 0.01.

Free‐text responses containing comments or concerns about completing the kit or the results were recorded for 614/2654 participants (23.1%) and were coded into 20 themes (Supporting Information S1: Tables 1–3). The most common themes were ‘ease of test’ (*n* = 81/614; 13.2%) and ‘a positive self‐sampling experience’ (*n* = 55/614; 9.0%). Women described self‐sampling as being easy to carry out and a straightforward process, with some women attributing this to clear instructions: ‘*it was so easy! Unbelievable – it's cured my fear & bad experiences! Thank you for developing the self‐test. I could not believe how painless it was. I truly believe its healed years of fear surrounding it. Wow I have more confidence in my own body as well. Thank you!*’

Not all women found the sample collection straightforward. The fourth most endorsed theme, ‘issues using the self‐sampling device’ (*n* = 38/614; 6.2%), highlighted some of the problems that women faced. Some women reported issues trying to insert the swab up to the red line (a marker that is specific to the FLOQswab 552C.80 device), as per the instructions (‘I *found it extremely difficult to insert the swab up to red line, mine was nowhere close to reaching red line an inch & half out*’) and other women, from across different age groups, reported some pain or bleeding (*‘I had some pain and there seems to be some bleeding*’) (*n* = 33/614; 5.4%). In addition, some women who had previously faced physiological barriers to a clinician‐taken test (e.g., vaginal atrophy or vaginismus) described finding it hard to collect a sample themselves (*n* = 18/614; 2.9%).

Other women were worried about test accuracy (*n* = 49/614; 8.0%), expressed a lack of confidence in kit completion (*n* = 34/614; 5.5%) or drew comparisons between self‐sampling and traditional clinician screening (*n* = 36/614; 5.9%). Interestingly, some associated the absence of pain or discomfort with a lack of confidence in completion: ‘*I'm just concerned people say it's uncomfortable so since mine wasn't, I worry it's not as thorough*’.

Another theme was ‘hygiene and menstruation’ (*n* = 10/614; 1.6%), with some women unsure whether they could sample during their period or whether they should wash before taking the test. A few proposed ways to increase their confidence in kit completion (‘Self‐sampling recommendations’: *n* = 11/614; 1.8%), including a practice swab, a GP tutorial‐style video sent in advance and streamlining the contents of the self‐sampling pack to avoid confusion (Supporting Information S1: Table 2).

### 3.3 Study Outcomes ‐Future Preferences for Screening/Self‐Sampling

#### 3.3.1 Preferred Screening Method

If given a choice, most women said they would opt for self‐sampling in the future (71.3%), with only a tenth of responders (10.4%) preferring a clinician‐taken test (Table 3). In the unadjusted analyses, age, ethnicity and screening status were significantly associated with women's screening preferences. In the fully adjusted model, most ethnic minority groups (Mixed: adjusted odds ratio [AOR] = 2.0, 95% CI: 1.2−3.2; Asian: AOR = 1.7, 95% CI: 1.3−2.2; Other Ethnic groups: AOR = 1.7, 95% CI: 1.2−2.6) and women from Other White backgrounds (AOR = 2.2, 95% CI: 1.7−2.8) were more likely to opt for self‐sampling, compared to White British women. Very overdue screeners (AOR = 2.0, 95% CI: 1.5−2.6) and those that had never attended screening before (AOR = 1.5, 95% CI: 1.2−1.8) were also at greater odds of selecting self‐sampling compared to overdue screeners (Table 4). The fully adjusted model was a good fit (*χ*²[15] = 113.59, *p* < 0.001), explaining 6.6% of the variance in screening preferences.

**Table 3:** Future preferences for screening/self‐sampling (*n* = 2654).

	*N*	Proportion (row %)
*Preferred screening method*		
Self‐sampling	1892	71.3
Clinician screening	277	10.4
No preference	403	15.2
Missing	82	3.1
*Preferences for urine v s. vaginal self‐sampling*		
Urine	1111	41.9
Vaginal	410	15.4
No preference	1038	39.1
Missing	95	3.6
*Preferences for receiving a self‐sampling kit*		
In the post	1743	65.7
In person at the GP surgery	285	10.7
No preference	541	20.4
Missing	85	3.2
*Preference for receiving results* a		
Willing to receive by text message	1671	63.0
Willing to receive by letter	1747	65.8
Willing to receive by email	1829	68.9
Missing	52	2.0

^a^ For the ‘preference for receiving results’ variable, women were asked to tick all options that applied. Therefore, each line item has been calculated as a proportion of all questionnaire responders (*n* = 2654).

**Table 4:** Women's future preferences for screening/self‐sampling (self‐sampling vs. clinician screening or no preference).

	Proportion (row %)			Logistic regression (self‐sampling vs. clinician screening or no preference [Ref])	
	Self‐sampling	Clinician screening	No preference	OR (95% CI)	AOR (95% CI)
*Age*					
25−34 years (*n* = 976)	68.5	12.5	19.0	Ref	Ref
35−44 years (*n* = 672)	74.0	11.4	14.6	1.30 (1.05−1.62)	1.24 (0.98−1.56)
45−54 years (*n* = 524)	77.5	9.9	12.6	1.58 (1.24−2.02)*	1.38 (1.05−1.80)
55−65 years (*n* = 400)	80.0	6.5	13.5	1.84 (1.39−2.43)*	1.51 (1.10−2.07)
*Ethnicity*					
White (*n* = 654)	64.4	13.6	22.0	Ref	Ref
White ‐ Other (*n* = 787)	81.6	6.9	11.5	2.45 (1.93−3.12)*	2.19 (1.71−2.82)*
Mixed/multiple ethnic groups (*n* = 110)	77.3	13.6	9.1	1.88 (1.17−3.02)*	1.97 (1.22−3.19)*
Asian (*n* = 596)	75.7	10.4	13.9	1.72 (1.35−2.20)*	1.70 (1.32−2.18)*
Black (*n* = 174)	66.7	13.8	19.5	1.11 (0.78−1.58)	1.07 (0.75−1.54)
Other ethnic group (*n* = 154)	75.3	13.0	11.7	1.69 (1.13−2.52)	1.74 (1.16−2.62)*
*Socioeconomic status (IMD)*					
Q1 (most deprived) (*n* = 449)	73.0	12.7	14.3	0.93 (0.63−1.37)	1.12 (0.74−1.68)
Q2 (*n* = 1074)	73.2	11.2	15.6	0.94 (0.66−1.33)	1.15 (0.80−1.67)
Q3 (*n* = 540)	73.1	9.3	17.6	0.93 (0.64−1.36)	1.10 (0.75−1.64)
Q4 (*n* = 314)	76.1	9.2	14.7	1.09 (0.72−1.66)	1.24 (0.80−1.92)
Q5 (least deprived) (*n* = 188)	74.5	10.1	15.4	Ref	Ref
*Mode of distribution*					
Mailed (*n* = 1266)	71.6	11.4	17.0	Ref	Ref
Opportunistic (*n* = 1306)	75.5	10.1	14.4	1.22 (1.03−1.46)	1.06 (0.88−1.29)
*Screening status*					
Overdue (up to 24 months since due date) (*n* = 1274)	68.3	13.1	18.6	Ref	Ref
Very overdue (> 24 months since due date) (*n* = 596)	84.1	5.7	10.2	2.45 (1.91−3.14)*	1.97 (1.50−2.59)*
Never attended (*n* = 695)	74.4	10.6	15.0	1.35 (1.10−1.66)*	1.45 (1.16−1.80)*

*Note:* All variables were included in the fully adjusted model.

Abbreviations: AOR, adjusted odds ratios; OR, unadjusted odds ratios; Ref, reference group.

* *p *< 0.01.

#### 3.3.2 Preferred Self‐Sampling Test

When asked about their preference if urine testing was an option, more women opted for a urine self‐sample (41.9% vs. 15.4% for vaginal), but a substantial proportion reported no preference (39.1%). In the unadjusted model, age and ethnicity were significantly associated with preferences. The fully adjusted odds of selecting urine self‐sampling (compared to vaginal or no preference) were higher for women over 35 years (35–44‐year‐olds: AOR = 1.4, 95% CI: 1.1–1.8; 45–54‐year‐olds: AOR = 1.6, 95% CI: 1.3–2.1; 55–64‐year‐olds: AOR = 2.2, 95% CI: 1.7–2.9), compared to the youngest age group (25–34‐year‐olds). Women from an Asian (AOR = 2.3, 95% CI: 1.8–2.8) or Black (AOR = 2.0, 95% CI: 1.4–2.8) background and those from Other Ethnic groups (AOR = 2.2, 95% CI: 1.5–3.1) were also more likely to opt for a urine test, compared to White British women (Table 5). The fully adjusted model was a good fit (*χ*²[15] = 120.12, *p* < 0.001), explaining 6.4% of the variance in women's preferred self‐sampling test.

**Table 5:** Women's preferences for HPV urine self‐sampling versus HPV vaginal self‐sampling or no preference.

	Proportion (row %)			Logistic regression (urine sample vs. vaginal sample or no preference [Ref])	
	Urine self‐sampling	Vaginal self‐sampling	No preference	OR (95% CI)	AOR (95% CI)
*Age*					
25−34 years (*n* = 975)	36.8	21.1	42.1	Ref	Ref
35−44 years (*n* = 667)	44.7	15.3	40.0	1.39 (1.13−1.69)*	1.41 (1.14−1.75)*
45−54 years (*n* = 518)	48.1	12.7	39.2	1.59 (1.28−1.97)*	1.64 (1.29−2.08)*
55−65 years (*n* = 399)	51.4	9.0	39.6	1.81 (1.43−2.30)*	2.21 (1.69−2.89)*
*Ethnicity*					
White (*n* = 655)	34.5	18.3	47.2	Ref	Ref
White ‐ Other (*n* = 776)	38.0	16.0	46.0	1.16 (0.94−1.45)	1.01 (0.80−1.27)
Mixed/multiple ethnic groups (*n* = 108)	47.2	12.0	40.8	1.70 (1.13−2.56)	1.69 (1.11−2.57)
Asian (*n* = 593)	54.1	14.9	31.0	2.24 (1.78−2.81)*	2.25 (1.79−2.84)*
Black (*n* = 173)	53.8	14.4	31.8	2.21 (1.57−3.10)*	2.01 (1.42−2.83)*
Other ethnic group (*n* = 155)	53.5	16.8	29.7	2.19 (1.54−3.12)*	2.17 (1.52−3.12)*
*Socioeconomic status (IMD)*					
Q1 (most deprived) (*n* = 443)	45.6	15.3	39.1	1.07 (0.76−1.51)	1.11 (0.77−1.59)
Q2 (*n* = 1072)	44.7	15.9	39.4	1.03 (0.76−1.41)	1.04 (0.75−1.44)
Q3 (*n* = 538)	40.3	17.7	42.0	0.86 (0.62−1.21)	0.90 (0.63−1.27)
Q4 (*n* = 309)	41.1	15.2	43.7	0.89 (0.62−1.29)	0.92 (0.63−1.35)
Q5 (least deprived) (*n* = 189)	43.9	14.3	41.8	Ref	Ref
*Mode of distribution*					
Mailed (*n* = 1263)	44.0	15.1	40.9	Ref	Ref
Opportunistic (*n* = 1296)	42.8	17.0	40.2	0.95 (0.82−1.11)	0.95 (0.80−1.12)
*Screening status*					
Overdue (up to 24 months since due date) (*n* = 1264)	41.7	17.1	41.2	Ref	Ref
Very overdue (> 24 months since due date) (*n* = 592)	47.8	13.2	39.0	1.28 (1.05−1.56)	1.10 (0.88−1.37)
Never attended (*n* = 695)	42.9	16.2	40.9	1.05 (0.87−1.27)	1.10 (0.90−1.34)

*Note:* All variables were included in the fully adjusted model.

Abbreviations: AOR, adjusted odds ratios; OR, unadjusted odds ratios; Ref, reference group.

* *p *< 0.01.

Further exploratory analyses identified significant associations between confidence in completion, trust in the results and preferred self‐sampling test. Women who reported lower confidence in using the YouScreen self‐sampling kit were significantly more likely to prefer urine self‐sampling in the future (63.3% among those with low confidence vs. 39.9% in those with high confidence). In addition, those reporting lower trust in the YouScreen results were at greater odds of preferring a urine‐based test (53.4% among those with low trust vs. 41.0% in those with high trust).

#### 3.3.3 Preferences for Receiving a Self‐Sampling Kit and Results

Most women said they would prefer to receive their self‐sampling kit in the post (65.7%) but preferences varied between demographic groups. Women from areas of higher deprivation and those from Asian, Black or Other ethnic groups were more likely to prefer GP collection, whereas younger women and those from Mixed/multiple backgrounds were more likely to opt for the post (see Supporting Information S1: Table 4).

Similar proportions of women said they would be willing to receive their screening results by text (63.0%), letter (65.8%) or email (68.9%). However, preferences varied between demographic groups (see Supporting Information S1: Table 5).

## 4 Discussion

This is the first large‐scale study to explore the experiences and preferences of previous screening non‐attenders completing a vaginal HPV self‐sample for cervical screening. In this socio‐economically and ethnically diverse population in England, confidence in test completion and accuracy were high and strong preferences were expressed for future self‐sampling.

Lack of confidence in self‐sampling kit completion and fears around test accuracy are well documented [12, 13, 14]. This study reported higher levels of confidence and trust compared to other UK‐based studies, although there is heterogeneity in design, settings and patient groups [27, 28, 29]. One explanation for high confidence could be the reassuring wording that was used in participant materials to increase confidence (e.g., ‘You can do it’). Alternatively, confidence may have increased due to widespread experience of completing self‐test kits for Covid‐19 [30]. Although trust and confidence were high across all subgroups, we found that Asian women reported significantly lower confidence and trust in the results. These findings mirror other studies that have explored ethnic disparities in attitudes to HPV vaginal self‐sampling [28, 31, 32]. Such studies have shown that Bangladeshi and Pakistani women are more frightened to carry out self‐collection and would have greater confidence in a professional doing the test. Further research is needed to understand the factors driving these attitudes and whether targeted interventions could help certain ethnic minority populations to make informed choices if self‐sampling were offered to them. We also found (as part of the free‐text comments) that some women lacked confidence in their completion of self‐sampling because they did not experience pain or discomfort when using the kit. Despite the fact that this was addressed in the information materials, some women still expected the test to be painful, possibly because they did not read the instructions carefully and knew that clinician screening can be uncomfortable.

Our research found that three‐quarters of women had high intentions to go for a follow‐up test if needed, but in fact, 90% of women in this survey sample who tested positive for HPV attended a follow‐up appointment, including women from Asian backgrounds who had reported significantly lower intentions of going for a clinician‐taken test [25]. This finding was unexpected because it is common to see an ‘intention‐behaviour gap’ (such that not all positive intentions translate into behaviour) [33], whereas our results show the opposite—that even those with low intentions attended. The unexpected distribution of responses on this item could have been due to a type‐setting error on the questionnaire, which led to the response options for this item being reversed compared to the preceding items for confidence and trust. As a result, participants who did not read the responses carefully may have ticked ‘1’ on the scale when they meant to tick ‘5’. Further work is needed to corroborate whether an inverse intention‐behaviour gap exists, but it is possible that the benefits of further investigation or the fear of cervical cancer can override previous barriers to traditional screening among women who receive a positive HPV result following self‐sampling. In addition, GP practices participating in the trial may have assisted or encouraged HPV‐positive women to book follow‐up appointments. High adherence to follow‐up is essential to ensure that self‐sampling participation is translated into clinical benefit [23]. Further qualitative research should explore how to support those who have previously refused a speculum examination if they require a follow‐up clinician‐taken test.

Most women who provided a free‐text comment about self‐sampling found the kit easy to use and reported a pleasant experience. However, it was apparent that some instructions were unclear (e.g., using the red line as a marker for insertion); some women experienced pain and bleeding; and others misunderstood the key differences between traditional screening and self‐sampling. Recommendations were proposed by a small number of women to improve future self‐sampling experiences (e.g., a swab to practice with, a GP tutorial video sent in advance of the kit and a streamlined self‐sampling pack). Including a lengthy participant information sheet and other study materials was a prerequisite for the trial. However, it is likely that future self‐sampling packs would contain fewer materials which would address this issue. In addition to the recommendations, other women had specific questions about the procedure. We have used these to develop a list of frequently asked questions which could be considered when developing future self‐sampling participant‐facing materials (see Supporting Information S1: Table 3).

We identified strong future preferences for self‐sampling (preferred by 71%), in line with other studies showing most non‐attenders (65%−88%) would prefer self‐collection [34, 35, 36, 37]. This suggests that offering this option in a national programme could increase screening uptake in non‐attenders. Preferences for self‐sampling were higher in certain ethnic groups (Asian, Mixed, Other White and Other Ethnic groups). Embarrassment, language difficulties, poor experiences with health professionals and specific cultural issues (e.g., female genital mutilation) [11, 38] are well‐established barriers to a clinician‐taken test in ethnic minority groups and may go some way to explaining preferences in these groups. In addition, women who were the most overdue for their test (> 24 months) and those that had never previously attended were more likely to prefer self‐sampling. One explanation could be that these women had not attended due to their dislike of the procedure (rather than simply not having got round to it). However, future research is needed to test this hypothesis.

More women preferred urine self‐sampling to vaginal self‐sampling, consistent with the findings of other studies [16, 17]. This has previously been attributed to the ease of testing [39]. However, we need to be careful when interpreting these results because women who completed this survey were not told that urine self‐testing is potentially less sensitive to detect high‐grade cervical disease, compared to vaginal self‐collection [40]. In addition, women could only select ‘urine’, ‘vaginal’ or ‘no preference’ response options for this question. Women who had already said that they would prefer a health professional to carry out their test in the future were still asked to choose between self‐sampling methods, which may have skewed the results if their preferences were different from those preferring self‐sampling. Additional analysis found no significant difference in responses between women who preferred self‐sampling and those who would choose clinician screening, which gives us confidence that this finding is robust.

We also asked women about how self‐sampling kits should be offered and found a preference for receiving the kit in the post compared to collecting from their GP surgery. Evidence from randomised trials has shown that uptake is higher when self‐sampling kits are mailed to women, compared to opt‐in strategies (where women must request their kits) [3, 19]. However, we need to be mindful that this study was carried out at the height of the Covid‐19 pandemic, which may have influenced women's preferences (i.e., this option supported social distancing measures and individuals were used to receiving Covid‐19 testing kits in the post). The YouScreen trial also demonstrated that offering kits opportunistically in primary care could be more effective than mailed kits (66% return rate for opportunistic kits vs. 13% for mailed) [25]. The opportunistic offer has several benefits: it provides an opportunity for GP endorsement, which has been shown to increase screening participation [41]; women are able to ask questions about the procedure, which may overcome previous barriers to clinician screening; and those who choose to complete a kit at the surgery avoid the logistics of returning their sample by post. Interestingly, the Australian cervical screening programme, which now offers a choice of cervical screening, had required women to carry out their tests at the surgery if they opted for self‐collection [42]. An advantage of this approach is that healthcare professionals are available to support women in obtaining their sample if they face difficulties.

Finally, we found similar levels of acceptability for distributing results by letter, text and email, although there were differences between demographic groups (see Supporting Information S1: Table 5). It is important that the screening programme considers the best way to deliver test results as this can help to reduce anxiety and influence future screening behaviours [43]. Our study shows that preferences for self‐sampling vary, which supports offering a choice of test and highlights the importance of considering women's preferences for receiving a kit and their results.

### 4.1 Study Strengths and Limitations

A key strength of this study was the inclusion of an ethnically and socially diverse sample; something that is often hard to achieve. This enabled us to look at demographic differences in self‐sampling experiences and future screening preferences with a degree of confidence. However, a key limitation was that only kit‐completers returned a questionnaire, which means our findings reflect the views of previous non‐attenders who chose to complete self‐collection. Although we tried to collect data from participants who did not complete a kit, our attempts to encourage these women to return a questionnaire were unsuccessful, despite the inclusion of specific questions for this group. This reflects the difficulty of engaging nonattender populations rather than any study design flaw. Alternative methodologies may have been more successful at encouraging these women to respond (e.g., a specific follow‐up questionnaire to kit non‐completers or greater signposting to the survey as part of the YouScreen pack). Future studies should consider alternative strategies to elicit the views of this important group of women. We also had a lower response rate than was anticipated based on previous studies [12, 23, 29], which limits the generalisability of our findings, particularly as survey respondents differed from kit‐completers in terms of ethnicity. In addition, we only recruited women from urban areas of London. We acknowledge that our results may have differed in rural locations.

## 5 Conclusion

This study is the first to explore women's experiences of self‐sampling, and future screening preferences, in a large and diverse nonattender population. Our research found that most women felt confident completing the kit, found it easy to use and had high trust in the test results. However, experiences varied between ethnic groups and some women highlighted difficulties using the kit. Self‐sampling was the preferred choice for future screening for most women, but it was not a universal preference; and therefore, offering a choice is likely to be important.

## Author Contributions

**Hannah Drysdale:** writing–original draft, writing–review and editing, formal analysis. **Laura A. V. Marlow:** supervision, writing–review and editing, methodology, writing–original draft, formal analysis. **Anita Lim:** conceptualisation, funding acquisition, writing–review and editing, supervision, methodology, data curation, project administration. **Jo Waller:** supervision, funding acquisition, conceptualisation, methodology, writing–review and editing, writing–original draft.

## Ethics Statement

Ethical approval for this questionnaire study was granted as part of the YouScreen trial. The trial (National Institute for Health Research ID: 41934) received approval from the South Birmingham Research Ethics Committee (20/WM/0120), Integrated Research Application System (IRAS) (ID 264776), the Confidentiality Advisory Group (20/CAG/0086) and the CSP Research Advisory Committee (CSP‐RAC‐032).

## Consent

Prior consent for the trial was not sought as the self‐sampling offer was made as a clinical service with approval from the Confidentiality Advisory Group. However, a study‐specific mechanism enabled women to opt out of receiving mailed kits and data sharing. Participants provided informed consent before taking part in the trial (implicit upon return of a self‐sample). Consent for the questionnaire study was implicitly granted through the return of the questionnaire.

## Conflicts of Interest

A. L. declares in‐kind support from Copan Italia S.p.A (provision of the 552C.80 FLOQSwab for the YouScreen study; receipt of honorarium for a lecture from Roche [Dec 2021]; and travel and accommodation to attend an expert meeting from Copan [Dec 2022]).

## Supporting information

Supporting information.

## Data Availability

The questionnaire and statistical analysis plan are available on Open Science Framework (OSF) (https://osf.io/b3m6h/). The dataset will be uploaded once all manuscripts have been published.
