# Supplementary material for: Experiences of Self‐Sampling and Future Screening Preferences in Non‐Attenders Who Returned an HPV Vaginal Self‐Sample in the YouScreen Study: Findings From a Cross‐Sectional Questionnaire
Source: Health Expect. 2024 Jul 2;27(4):e14118. doi: 10.1111/hex.14118 (PMC11217896; doi:10.1111/hex.14118)
Supplement: Supplementary file 1 — Supporting information. [file HEX-27-e14118-s001.docx]

**Supplementary materials – Free text responses (Tables S1-S3)**

**Table S1.** *Themes identified as part of the evaluation of women’s free-text responses on self-sampling*

|  | *Frequency* | *%* |
| --- | --- | --- |
| Ease of test | 81 | 13.2 |
| A positive self-sampling experience | 55 | 9.0 |
| Concerns about test accuracy | 49 | 8.0 |
| Issues using the self-sampling device | 38 | 6.2 |
| Comparing self-sampling and clinician screening | 36 | 5.9 |
| Confidence in kit completion | 34 | 5.5 |
| Pain or bleeding when self-sampling | 33 | 5.4 |
| Other | 29 | 4.7 |
| Physiological issues with self-sampling | 18 | 2.9 |
| Self-sampling overcomes barriers | 12 | 2.0 |
| Self-sampling results | 12 | 2.0 |
| Self-sampling recommendations | 11 | 1.8 |
| Hygiene and menstruation | 10 | 1.6 |
| Comparing self-sampling and clinician sampling | 11 | 1.1 |
| The logistics of returning a sample | 7 | 1.1 |
| Follow-up post self-testing | 4 | 0.7 |
| Help from others when self-sampling | 2 | 0.3 |
| Self-sampling supports marginalised groups | 2 | 0.3 |
| The impact of Covid-19 on self-testing | 1 | 0.2 |
| Invalid responses | 270 | 44.0 |

*Note.* Percentages are calculated as a proportion of the women that provided a free-text response (n=614).

**Table S2.** *Self-sampling recommendations as part of the free-text responses*

Some questionnaire responders proposed ways that self-sampling could be improved. These comments were coded under the theme “Self-sampling recommendations” (n=11/614, 1.8%).

| Self-sampling recommendations |
| --- |
| Diagrams to aid completion |
| GP to send a tutorial-style video in advance of test completion |
| The patient information booklet should make it clear whether women can do the test while menstruating |
| Would like two kits (i.e., for practice or if a mistake is made) |
| Supply a sticker/tape to seal the tube after the self-sampling test |
| Patient information booklet needs to be online in English |
| Suggestion to streamline kit contents as was confusing |
| Make it clear that postage is pre-paid |
| Further information on why there is a five-day limit to the test and how does this work if menstruating |
| Suggestion for clearer instructions |
| The red line was hard to see - suggest a ridge or bump on the swab so you can feel where to insert it to |

**Table S3**. *Frequently asked questions for future self-sampling participant materials*

Some women had specific questions about the self-sampling kit or procedure, and others showed a lack of understanding about the differences between self-collection and a clinician-taken test. Examples of questions and queries that were raised in the free-text responses have been included in Table S3 (and sit outside of the coding framework) and could be considered when introducing self-sampling into a national screening programme.

| Frequently asked questions |
| --- |
| Is it a problem if I accidently drop the swab on the floor?  How is self-sampling effective if the swab does not reach the cervix to collect a sample of cervical cells (like in a clinician-taken test)?  If a self-sample is all you need to do, why do doctors/nurses have to use the speculum in the surgery or health centre? |
| Can you still do the self-sample if you have thrush or another yeast infection? |
| Should you have a bath before taking the test? |
| Does there need to be liquid in the self-sampling container? (Indicates reference to covid-19 lateral flow tests)  What would happen if the test came back as an inadequate sample? Would another swab be sent for me to complete? |

**Supplementary materials - Preferences for self-sampling offer (Tables S4-S5)**

**Table S4.** *Women’s preferences for receiving a self-sampling kit in the future*

| Proportion (row %) |  | | |
| --- | --- | --- | --- |
|  | Post | GP | No preference |
| *Age*  25-34 years (n=978)  35-44 years (n=672)  45-54 years (n=520)  55-65 years (n=399) | 68.6  71.7  66.5  61.1 | 11.0  9.7  12.9  11.3 | 20.4  18.6  20.6  27.6 |
| *Ethnicity*  White (n=657)  White - Other (Irish and Other) (n=779)  Mixed/multiple ethnic groups (n=106)  Asian or Asian British (n=596)  Black, Black British, Caribbean, or African (n=176)  Other ethnic group (n=156) | 66.7  70.5  79.2  69.5  54.0  68.6 | 9.0  8.0  6.6  13.4  18.7  15.4 | 24.3  21.5  14.2  17.1  27.3  16.0 |
| *Socioeconomic Status (IMD)*  Q1 (most deprived) (n=448)  Q2 (n=1073)  Q3 (n=537)  Q4 (n=311)  Q5 (least deprived) (n=192)  *Mode of distribution*  Mailed (n=1265)  Opportunistic (n=1304)  *Screening status*  Overdue (up to 24m since due date) (n=1264)  Very overdue (>24m since due date) (n=597)  Never attended (n=700) | 63.9  67.8  65.9  73.6  74.0  80.4  55.7  70.0  66.0  65.6 | 15.6  11.0  10.6  7.7  7.8  5.2  16.8  10.7  12.1  11.0 | 20.5  21.2  23.5  18.7  18.2  14.4  27.5  19.3  21.9  23.4 |

**Table S5.** *Women’s preferences for receiving their screening results.*

| Proportion (row %) |  | | |
| --- | --- | --- | --- |
|  | Text message | Letter | Email |
| *Age*  25-34 years (n=1005)  35-44 years (n=691)  45-54 years (n=536)  55-65 years (n=422) | 69.7  65.0  59.3  48.3 | 59.5  68.3  70.5  70.9 | 74.9  70.6  65.3  56.4 |
| *Ethnicity*  White (n=675)  White - Other (Irish and Other) (n=802)  Mixed/multiple ethnic groups (n=112)  Asian or Asian British (n=614)  Black, Black British, Caribbean, or African (n=182)  Other ethnic group (n=162) | 59.9  67.3  60.7  64.5  61.0  62.3 | 63.0  70.6  71.4  63.0  63.2  67.3 | 74.2  74.1  72.3  63.4  61.0  60.5 |
| *Socioeconomic Status (IMD)*  Q1 (most deprived) (n=463)  Q2 (n=1105)  Q3 (n=564)  Q4 (n=319)  Q5 (least deprived) (n=195)  *Mode of distribution*  Mailed (n=1307)  Opportunistic (n=1347)  *Screening status*  Overdue (up to 24m since due date) (n=1313)  Very overdue (>24m since due date) (n=617)  Never attended (n=716) | 61.8  63.7  63.1  62.1  63.6  60.7  65.1  65.7  61.6  59.4 | 65.4  66.7  65.1  66.8  62.1  67.5  64.2  65.8  70.5  61.7 | 60.7  66.8  72.7  74.9  80.5  65.3  72.4  69.8  61.8  73.7 |

*Note.* Women were asked to tick all options that applied. Therefore, each line item has been calculated as a proportion of all questionnaire responders (n=2654).
